# *Alanine-glyoxylate aminotransferase 2* (*AGXT2*) Polymorphisms Have Considerable Impact on Methylarginine and β-aminoisobutyrate Metabolism in Healthy Volunteers

**DOI:** 10.1371/journal.pone.0088544

**Published:** 2014-02-24

**Authors:** Anja Kittel, Fabian Müller, Jörg König, Maren Mieth, Heinrich Sticht, Oliver Zolk, Ana Kralj, Markus R. Heinrich, Martin F. Fromm, Renke Maas

**Affiliations:** 1 Institute of Experimental and Clinical Pharmacology and Toxicology, Friedrich-Alexander-Universität Erlangen-Nürnberg, Erlangen, Germany; 2 Institute of Biochemistry, Friedrich-Alexander-Universität Erlangen-Nürnberg, Erlangen, Germany; 3 Department of Chemistry and Pharmacy, Friedrich-Alexander-Universität, Erlangen-Nürnberg, Erlangen, Germany; Rikagaku Kenkyūsho Brain Science Institute, Japan

## Abstract

Elevated plasma concentrations of asymmetric (ADMA) and symmetric (SDMA) dimethylarginine have repeatedly been linked to adverse clinical outcomes. Both methylarginines are substrates of alanine-glyoxylate aminotransferase 2 (AGXT2). It was the aim of the present study to simultaneously investigate the functional relevance and relative contributions of common *AGXT2* single nucleotide polymorphisms (SNPs) to plasma and urinary concentrations of methylarginines as well as β-aminoisobutyrate (BAIB), a prototypic substrate of AGXT2. In a cohort of 400 healthy volunteers ADMA, SDMA and BAIB concentrations were determined in plasma and urine using HPLC-MS/MS and were related to the coding *AGXT2* SNPs rs37369 (p.Val140Ile) and rs16899974 (p.Val498Leu). Volunteers heterozygous or homozygous for the *AGXT2* SNP rs37369 had higher SDMA plasma concentrations by 5% and 20% (p = 0.002) as well as higher BAIB concentrations by 54% and 146%, respectively, in plasma and 237% and 1661%, respectively, in urine (both p<0.001). ADMA concentrations were not affected by both SNPs. A haplotype analysis revealed that the second investigated *AGXT2* SNP rs16899974, which was not significantly linked to the other *AGXT2* SNP, further aggravates the effect of rs37369 with respect to BAIB concentrations in plasma and urine. To investigate the impact of the amino acid exchange p.Val140Ile, we established human embryonic kidney cell lines stably overexpressing wild-type or mutant (p.Val140Ile) AGXT2 protein and assessed enzyme activity using BAIB and stable-isotope labeled [^2^H_6_]-SDMA as substrate. *In vitro*, the amino acid exchange of the mutant protein resulted in a significantly lower enzyme activity compared to wild-type AGXT2 (p<0.05). *In silico* modeling of the SNPs indicated reduced enzyme stability and substrate binding. In conclusion, SNPs of *AGXT2* affect plasma as well as urinary BAIB and SDMA concentrations linking methylarginine metabolism to the common genetic trait of hyper-β-aminoisobutyric aciduria.

## Introduction

In various clinical studies elevated plasma concentrations of endogenously formed symmetric (SDMA) and asymmetric (ADMA) dimethylarginine were identified as prospective and independent risk markers of cardiovascular diseases and mortality [1]–[3]. Largely based on animal experiments, an active role in disease progression and/or development has been suggested [4], [5]. Interference with L-arginine metabolism and signaling is commonly considered a (if not the) key mechanism. In addition, it was shown that both dimethylarginines compete with L-arginine for uptake into the cell by cationic amino acid transporter 1 (CAT1) [6] and that ADMA acts as an endogenous inhibitor of nitric oxide synthases [7], [8]. However, it has repeatedly been speculated that the interference of methylarginines with L-arginine metabolism and signaling may not be the only mechanism linking methylarginines with human disease [9]. Elevation of methylarginine concentrations may simply indicate structural or functional deficiencies of the metabolizing enzyme(s) dimethylarginine dimethylaminohydrolase (DDAH1 and DDAH2, which degrade ADMA) and alanine-glyoxylate aminotransferase 2 (AGXT2, which degrades ADMA and SDMA). These enzymes have further substrates (as detailed below) [10] and may be involved in alternative regulatory mechanisms [11]. Therefore, it is possible (but poorly investigated, so far), that these alternative substrates and functions may explain some of the adverse effects currently attributed to methylarginines.

In 1986 Ogawa and coworkers [12] showed for the first time in rats that Agxt2 metabolizes both dimethylarginines. A recent study linked SDMA plasma concentrations in humans with polymorphisms within the *AGXT2* gene [13]. Recently, further data were published indicating that a knockout of *Agxt2* in mice is associated with elevation of ADMA and SDMA plasma concentrations and that the *AGXT2* single nucleotide polymorphism (SNP) rs37369 (c.418G>A, p.Val140Ile) is associated with hypertension in humans [14]. However, ADMA and SDMA are not the only known substrates of AGXT2 resulting in two isomers of (dimethylguanidino)valeric acid (DMGV and DM’GV). In 1993, Agxt2 was found to be identical with an enzyme called “D-3-aminoisobutyrate-pyruvate aminotransferase” [10]. β-Aminoisobutyrate (BAIB), an end product of the pyrimidine metabolism, is an additional substrate of Agxt2. This was confirmed in a genome-wide association study published by Suhre et al. [15] linking the coding *AGXT2* SNP rs37369 with a higher urinary excretion of BAIB (hyper-BAIB aciduria), a heritable trait which first was described in 1951 [16].

So far, the functional role of AGXT2 has been investigated either with a focus on methylarginines or BAIB. It was the aim of the present study to shed more light on the interrelation of these analytes and their metabolism by AGXT2. Furthermore, the impact of *AGXT2* SNPs on BAIB plasma concentrations was not known. We therefore determined methylarginines and BAIB concentrations in plasma and urine of 400 healthy volunteers and related these to both investigated SNPs in the *AGXT2* gene (rs37369 and rs16899974: c.1492G>T, p.Val498Leu). The *in vivo* studies were complemented by *in silico* studies regarding both SNPs as well as *in vitro* studies for the *AGXT2* SNP rs37369.

## Materials and Methods

### 3.1 Study Protocol and Participants

The KPE19 (Klinische Pharmakologie Erlangen 19) study was initiated, organized and performed at the Institute of Experimental and Clinical Pharmacology and Toxicology, Friedrich-Alexander-Universität Erlangen-Nürnberg (Germany). Four hundred healthy volunteers were included in the study. The main study aim was the identification of subjects with genetic or phenotypic variants for subsequent pharmacogenetic and pharmacokinetic studies. The secondary aim was the correlation of concentrations of endogenous substances in blood or urine with genetic variants. The study was approved by the Ethics committee of the Friedrich-Alexander-Universität Erlangen-Nürnberg and all participants provided written informed consent. Healthy male and female volunteers between 18 and 60 years were eligible for inclusion. Excluded from participation were subjects with drug or alcohol abuse, concomitant use of medication (except for thyroid hormone substitution or hormonal contraception), excessive alcohol or caffeine consumption, intake of grapefruit or grapefruit juice within the last 2 days, excessive work-out within the last 10 days, pregnancy, a history of cancer within the last 5 years, current relevant disease, impaired kidney or liver function, blood loss ≥400 ml within the last 21 days or participants dependent on one of the investigators. A physician took a complete medical history from each participant. The mean±SD/median (25–75% percentile) fasting time prior to blood and urine sampling was 5.7±5.1/3.0 (2.0–11.0) h, respectively. Blood samples for DNA extraction were available from 400 subjects and were stored at −20°C until extraction. Plasma and serum samples were available from 395 and 390 subjects, respectively, and were stored at −20°C until analysis. Mid-stream urine was stored at −80°C until analysis. Urine samples were available from 400 subjects.

### 3.2 Chemicals

[^2^H_7_]-Labeled ADMA hydrochloride und [^2^H_7_]-labeled L-arginine hydrochloride were obtained from EURISO-TOP (Saint-Aubin, France). The isotope labeled BAIB standard ([^2^H_6_]-BAIB) was a kind gift of Dr. Ute Hofmann (Dr. Margarete Fischer-Bosch-Institute of Clinical Pharmacology, Stuttgart, Germany) [17]. The isotope labeled SDMA ([^2^H_6_]-SDMA) was obtained from Toronto Research Chemicals Inc. (Toronto, Canada). ADMA dihydrochloride and SDMA dihydrochloride were purchased from Enzo Life Sciences GmbH (Lörrach, Germany). L-Arginine was purchased from Sigma-Aldrich Chemie GmbH (Steinheim, Germany). Acetonitrile hypergrade for LC-MS and SUPRAPUR® formic acid (98%) were obtained from Merck (Darmstadt, Germany), and Baker Analyzed LC-MS-Reagent Water was from Mallinckrodt Baker B.V. (Deventer, Netherland). The AGXT2-dependent metabolite DM’GV was synthesized as described in detail in the figure S1.

### 3.3 Measurement of AGXT2 Substrate Concentrations via HPLC-MS/MS

L-Arginine, L-ADMA and L-SDMA concentrations were measured in plasma and urine of volunteers by HPLC-MS/MS (Agilent 1100 HPLC System [Agilent Technologies, Waldbronn, Germany]; API 4000 mass spectrometer [Applied Biosystems, Darmstadt, Germany]) as previously described with minor modifications [18], [19]. Separation was performed by using an EC 100/2 NUCLEOSHELL HILIC 2.7 µm column (Machery-Nagel, Düren, Germany). BAIB was measured in plasma and urine of volunteers as well as human embryonic kidney 293 (HEK) cell lysates by HPLC-MS/MS as previously described [20]. Creatinine measurement was performed at the central laboratory of Erlangen university hospital by use of the modified Jaffé method.

Peak area of [^2^H_6_]-DM’GV and DM’GV was determined by means of HPLC-MS/MS. For sample preparation a solution of approximately 10 µmol/L DM’GV was prepared in acetonitrile. 50 µl of this solution was mixed with 20 µl of the sample and 2 µl water. After centrifugation the supernatant was diluted 1∶10 with the eluent (20% water, 80% acetonitrile, 0.05% ammonium formiate; pH 4). Injection volume was 50 µl. For separation an EC 100/2 NUCLEODUR HILIC 2.7 µm column (Macherey-Nagel) combined with a guard column (EC4/2) was used. Chromatography was carried out isocratically at a flow rate of 0.4 ml/min. The mass transitions were m/z 202.1 to 71.10 for DM’GV and m/z 208.10 to 77.10 for [^2^H_6_]-DM’GV. The results were approved by the qualifiers m/z 202.1 to 70.10 for DM’GV and 208.10 to 70.10 for [^2^H_6_]-DM’GV.

### 3.4 Genotyping of *AGXT2* SNPs via TaqMan-based Real-time PCR Assay

DNA was extracted using the chemagic DNA Blood Kit (Chemagen Chemie, Baesweiler, Germany) according to the manufacturer’s instructions by the Institute of Human Genetics, Erlangen university hospital. Both probes for genotyping (rs37369 and rs16899974) were purchased from Applied Biosystems (Darmstadt, Germany). Quantitative real-time PCR was carried out using the ABI 7900HT system from Applied Biosystems according to the manufacturer’s instructions. For each sample 20 ng DNA was added to 3 µl of Taqman GTXpress MasterMix (Applied Biosystems), 1.85 µl RNase-free water and 0.15 µl TaqMan probe (40×). The cycle conditions for PCR were 95°C for 5 min, followed by 45 cycles of 95°C for 15 s and 60°C for 30 s.

### 3.5 Generation of Human Embryonic Kidney 293 Cell Lines Stably Overexpressing Human Wild-type and Mutant AGXT2

The human *AGXT2* cDNA (NM_031900) was cloned by a RT-PCR-based approach using kidney total RNA (ClonTech, Heidelberg, Germany) as template for single-strand cDNA synthesis via iScript Select cDNA Synthesis Kit (Bio-Rad, Munich, Germany). For amplification of full-length *AGXT2* cDNA, the following primer pair was used: forward 5′-TGA GTG GGA GAA ATG ACT CTA AT-3′ and reverse 5′-CTG ACA ATG TTA CTT AGC TCT TC-3′. The amplified fragment was cloned into the pCR2.1-TOPO vector via TOPO TA PCR Cloning Kit (Invitrogen, Karlsruhe, Germany) according to the manufacturer’s instructions.

Following sequencing by AGOWA (Berlin, Germany), base pair exchanges in comparison to the reference sequence (NM_031900) resulting in amino acid exchanges were corrected using the QuikChange Multi Site Directed Mutagenesis Kit (Agilent Technologies, Waldbronn, Germany). The corrected cDNA was subcloned into the expression vector pcDNA3.1(+) leading to the plasmid pAGXT2-WT.31. This plasmid was also used for cloning the mutant *AGXT2* cDNA containing the single-nucleotide polymorphism rs37369 (NM_031900.3). For the base pair exchange (c.418G>A) the QuikChange Multi Site Directed Mutagenesis Kit (Agilent Technologies, Waldbronn, Germany) was used leading to the plasmid pAGXT2-MUTrs37369. Correctness and orientation of the wild-type and mutant cDNA were verified by sequencing (AGOWA, Berlin, Germany).

HEK cells were then stably transfected with the empty expression vector pcDNA3.1(+) as control and with the two cloned plasmids pAGXT2-WT.31 and pAGXT2-MUTrs37369 using the Effectene Transfection Reagent Kit according to the manufacturer’s instructions (QIAGEN, Hilden, Germany). AGXT2 expression was determined in all three cell lines (HEK VC as vector control, HEK AGXT2 WT and HEK AGXT2 rs37369) using immunoblot analysis and immunofluorescence.

### 3.6 Immunoblot Analysis

For determination of AGXT2 protein expression in HEK cells (VC, AGXT2 WT and AGXT2 rs37369) immunoblot analysis was performed as described before with minor modifications [20]. Samples (5–20 µg of total protein) of cell lysates were prepared and separated by SDS-PAGE under reducing conditions on 10% polyacrylamide gels. Proteins were transferred to nitrocellulose membranes (Protran Nitrocellulose Transfer Membrane; Whatman, Dassel, Germany) using a tank blotting system from Bio-Rad (Munich, Germany) and probed with a rabbit polyclonal anti-human AGXT2 antibody (HPA037382; Sigma-Aldrich, Munich, Germany) at a dilution of 1∶500. For detection a horseradish peroxidase-conjugated goat anti-rabbit antibody (Sigma Aldrich, Munich, Germany) was used at a dilution of 1∶10000 and immunoreactive bands were visualized using ECL Western Blotting Detection Reagents from Amersham (GE Healthcare, Buckinghamshire, UK) and a Chemidoc XRS imaging system (Bio-Rad, Munich, Germany). To control sample loading, membranes were stripped and reprobed with a mouse monoclonal anti-human β-actin antibody (Sigma Aldrich, Munich, Germany) at a dilution of 1∶500. As secondary antibody a horseradish peroxidase-conjugated goat anti-mouse antibody (Dianova, Hamburg, Germany) was used at a dilution of 1∶10000.

To compare protein expression of AGXT2 between the cell lines a densitometric analysis was performed. After detection of AGXT2 and β-actin via immunoblot analysis the density average of every band was calculated using the Quantity One Software (Bio-Rad, Munich, Germany). The AGXT2 protein expression was normalized to β-actin. To figure out which concentration of the cell lysates results in the best signal for AGXT2 and β-actin within the linear detection range, different amounts of lysates (1 µg, 2.5 µg, 5 µg, 7.5 µg, 10 µg and 12.5 µg) of an overexpressing cell line were also separated by SDS-PAGE. Investigation of the immunoreactive bands after immunoblot analysis and creation of a calibration curve (total protein concentration versus density average of every band) indicated that the loading of 5 µg total protein of the cell lysates results in the best signal for AGXT2 as well as β-actin.

### 3.7 Immunofluorescence Microscopy

Expression and cellular localization of AGXT2 was analyzed in HEK cells (VC, AGXT2 WT and AGXT2 rs37369) using immunofluorescence staining. Cells were seeded and grown on coverslips (4×10^5^ cells/coverslip) placed in cell culture plates. AGXT2 expression was induced 24 h after seeding with 10 mM sodium butyrate. After additional 24 h cells were incubated with 50 nM MitoTracker Red CMXRos (Molecular Probes, Eugene, OR, USA) according to the manufacturer’s instructions. The human AGXT2 protein was detected using a rabbit polyclonal anti-human antibody (HPA037382; Sigma-Aldrich, Munich, Germany) at a dilution of 1∶400 followed by incubation with an Alexa Fluor 488 conjugated secondary antibody (Molecular Probes, Eugene, OR, USA) at a dilution of 1∶1000. For visualization the confocal microscope Axiovert 100 M (Carl Zeiss, Jena, Germany) and the Zeiss LSM Image Browser version 4.2.0.121 were used.

### 3.8 Enzyme Activity Assay

Cells were cultured as previously described [6]. Assays were performed on HEK cell lysates to measure differences between wild-type and mutant AGXT2 enzyme activity. Cells were grown in minimal essential medium (MEM) containing 10% heat-inactivated fetal bovine serum, 500 µg/ml geneticin, 100 U/ml penicillin and 100 µg/ml streptomycin at 37°C and 5% CO_2_. All cell culture supplements were purchased from Invitrogen (Karlsruhe, Germany). After 24 h AGXT2 expression was induced by 10 mM sodium butyrate and cells were incubated for additional 24 h. Cells were detached using trypsin (0.05%)-EDTA (0.02%), washed with PBS and resuspended in PBS containing 1 mM sodium pyruvate and 1 mM sodium glyoxylate as amino group acceptor, 0.1 mM pyridoxal-phosphate as co-factor for activation and 15 mM Tris buffer (pH 9.0). Cells were kept on ice and lysed via sonification (Sonifier B-12, Branson Sonic Power Company, Danbury, CT, USA). Protein concentrations of cell lysates were determined using a standard assay (BCA Protein Assay Reagent; Rockford, USA) according to the manufacturer’s instructions. To samples of 1 µg/µl protein 100 µM D,L-BAIB or 0.5 µM [^2^H_6_]-SDMA was added in a volume of 500 µl. Half of each sample (baseline of [^2^H_6_]-SDMA, [^2^H_6_]-DM’GV and D,L-BAIB concentrations) was heated at 95°C for 5 min to stop enzyme activity and stored at −20°C. The other half of each sample was incubated over night at 37°C and 500 rpm for 12 h, then heated at 95°C for 5 min and stored at −20°C. In each sample D,L-BAIB or [^2^H_6_]-SDMA and [^2^H_6_]-DM’GV concentrations were determined using HPLC-MS/MS.

### 3.9 *In silico* Modeling of the 3D Structures of the *AGXT2* Variants V140I (rs37369) and V498L (rs16899974)

The three-dimensional structure of AGXT2 was modeled based on the crystal structure of the homologous dialkylglycine decarboxylase (PDB code 1D7S) [21] using the program Modeller 9.9 [22]. The resulting model of the structure of wild-type AGXT2 exhibited a good local geometry and no steric hindrance. The V140I and V498L mutations were introduced into the structure using the program DeepView [23] by selecting the lowest-energy side chain rotamer. Clashes in the protein were analyzed with WhatCheck [24], and RasMol [25] was used for graphical presentation.

### 3.10 Statistical Analysis

Significant differences between analyte concentrations determined in plasma and urine of volunteers and different genotypes were analyzed by Kruskal-Wallis test (Dunn’s post-test) and Jonckheere-Terpstra trend-test. Independence and interactions of AGXT2 rs37369 and rs16899974 with respect to plasma and urinary concentrations of the analytes were analyzed by two-way ANOVA using log transformed data. When a two-way ANOVA identified both SNPs as significant corresponding nested ANOVAs were calculated to assess the effect of a SNP in the respective subgroups of the other SNP (to adjust for repeated testing in this case the significance level was lowered to <0.025 for the analysis of the subgroups). For correlation between ADMA, SDMA, BAIB and creatinine concentrations Spearman’s test was used. Comparison of AGXT2 expression and enzyme activity was performed using the unpaired 2-tailed Student’s t test. Unless stated otherwise, statistical significance was defined as a p value <0.05. Values are reported as mean ±SD and/or median (25–75% percentile). For the calculations, Prism 5 software (ver. 5.00 for Windows; GraphPad Software, San Diego, CA), STATA 12 (StataCorp LP, TX, USA) and SPSS Statistics (ver. 21; IBM, Ehningen, Germany) were used as appropriate. Linkage disequilibrium between AGXT2 rs37369 and rs16899974 was analyzed via Haploview 4.2 (Broad Institute, Cambridge, MA, USA).

## Results

### 4.1 Description of the Cohort

The demographic and biochemical characteristics of the cohort of 400 healthy volunteers of the KPE19 study are shown in tables 1 and 2.

**Table 1:** Demographic data of the KPE19 volunteer cohort.

	Characteristics	
**Sex**	**Female**	249 (62.25%)
	**Male**	151 (37.75%)
**Age (years)**		23 (21–26)*
**Ethnic group** †	**Caucasian**	394
	**Asian**	1
	**Latin American**	3
	**Others**	2

* Median value (25–75% percentile) is shown.

† self-reported.

**Table 2:** Biochemical data of the KPE19 volunteer cohort.

		Mean ±SD	Median (25–75% percentile)
**Plasma (µmol/L)** *	**SDMA**	0.42±0.07	0.41 (0.37–0.45)
	**ADMA**	0.38±0.07	0.37 (0.34–0.42)
	**L-arginine**	83.2±40.1	73.1 (57.7–98.4)
	**BAIB**	1.09±0.42	1.02 (0.80–1.21)
**Urine (µmol/g creatinine)** †	**SDMA**	37.0±7.8	36.4 (31.7–42.1)
	**ADMA**	33.3±9.2	31.6 (27.1–37.7)
	**L-arginine**	16.0±9.3	13.9 (10.8–18.6 )
	**BAIB**	90.1±130	54.0 (33.7–93.4)
**Creatinine**	**Serum (mg/dL)** ‡	0.8±0.1	0.8 (0.7–0.9)
	**Urine (mg/dL)** †	104±84	86 (37–144)
	**Clearance (ml/min)** ‡ §	127±28	123 (109–142)

Plasma and urinary concentrations were measured by HPLC-MS/MS.

* Plasma concentrations were determined for 395 volunteers.

† Urinary concentrations were measured for 400 volunteers.

‡ Serum creatinine concentrations and creatinine clearance were determined for 390 volunteers.

§ Cockcroft-Gault formula was used for calculation of creatinine clearance.

### 4.2 *AGXT2* SNPs - genotypes and Biochemical Correlates

**Figure 1:**
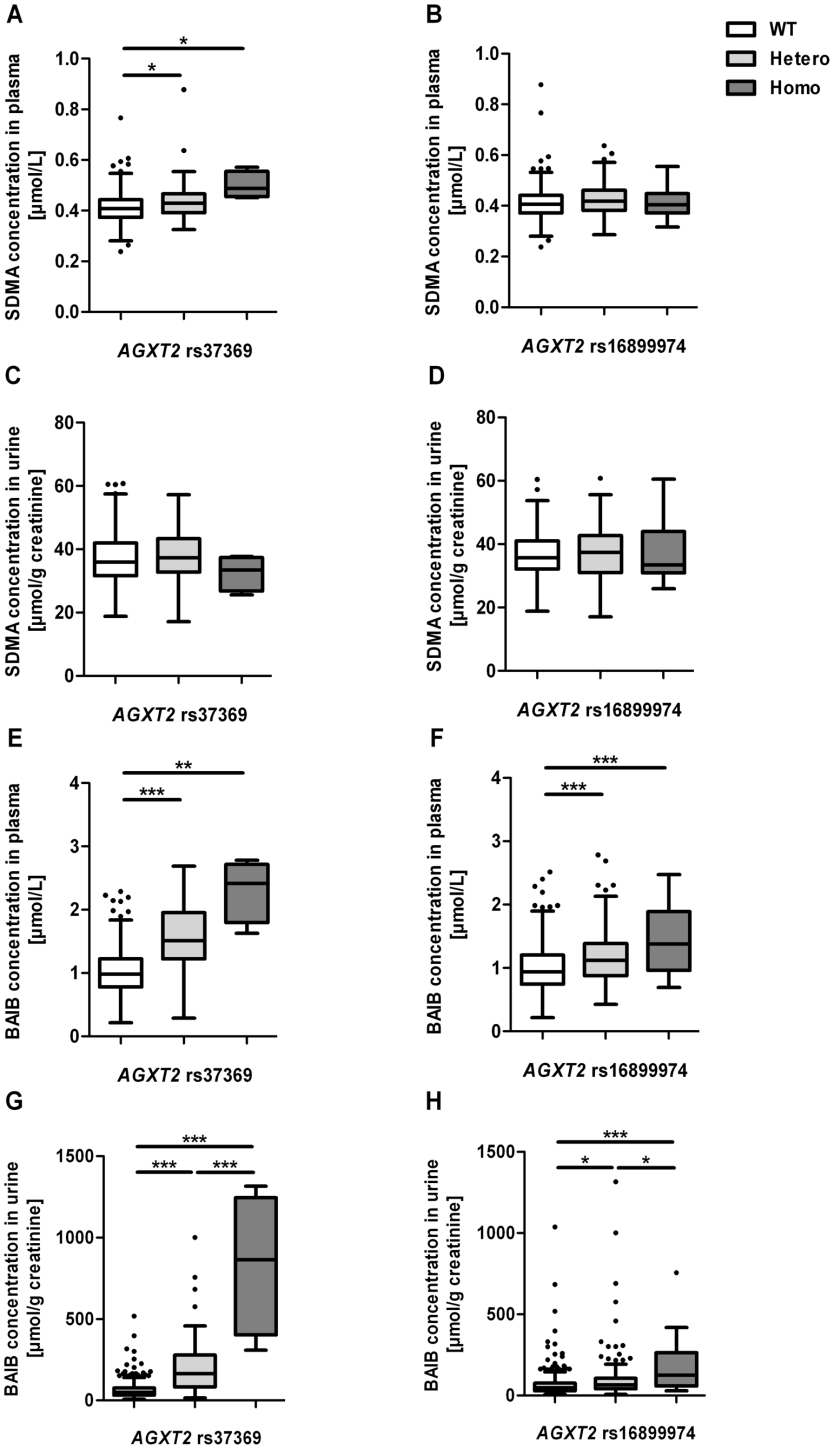
*AGXT2* SNPs and biochemical measures. The *AGXT2* SNP rs37369 was associated with significant differences in plasma SDMA (A), plasma (E) and urinary BAIB (G) concentrations, whereas *AGXT2* SNP rs16899974 was associated with significant differences in plasma (F) and urinary (H) BAIB concentrations. Plasma SDMA concentrations were not significantly different in case of rs16899974 (B), as well as urinary SDMA concentrations for both SNPs (C–D). Values are median ±1.5 IQR. Details on the underlying genotype distribution are shown in table 2 and in table S1. The Jonckheere-Terpstra trend-test (p<0.001 in all cases except for figure 1B–D) and Kruskal-Wallis test (Dunn’s post-test, * p<0.05, ** p<0.01, *** p<0.001) were used for statistical analysis. ADMA, asymmetric dimethylarginine; *AGXT2*, *alanine-glyoxylate aminotransferase 2*; BAIB, β-aminoisobutyrate; Hetero, heterozygous for minor allele; Homo, homozygous for minor allele; SDMA, symmetric dimethylarginine; WT (wild-type), homozygous for major allele.

Genotyping of the 400 KPE19 volunteers for the *AGXT2* SNP rs37369 (c.418G>A, p.Val140Ile) revealed a minor allele frequency (MAF) of 0.07 (table 3 and table S1). The *AGXT2* SNP rs37369 was associated with significantly higher plasma SDMA as well as plasma and urinary BAIB concentrations (table 3 and figure 1). Plasma SDMA concentrations were higher by 5% and 20% (Kruskal-Wallis test: p = 0.002), respectively, in volunteers heterozygous or homozygous for the minor allele of the *AGXT2* SNP rs37369, while the urinary SDMA concentrations were not significantly different. BAIB concentrations were higher by 54% and 146%, respectively, in plasma and by 237% and 1661%, respectively, in urine of volunteers heterozygous or homozygous for the minor allele (Kruskal-Wallis test: both p<0.001). Plasma and urinary concentrations of ADMA and L-arginine were not significantly affected by this SNP (table 3).

**Table 3:** *AGXT2* SNPs and biochemical measures.

		*AGXT2* rs37369				*AGXT2* rs16899974			
		WT (c.418 GG)	Hetero (c.418 GA)	Homo (c.418 AA)	p value	WT (c.1492 GG)	Hetero (c.1492 GT)	Homo (c.1492 TT)	p value
**Plasma (µmol/L)** *		n = 345	n = 46	n = 4		n = 239	n = 139	n = 17	
	**SDMA**	0.41 (0.37–0.44)	0.43 (0.39–0.47)	0.49 (0.46–0.56)	0.002	0.41 (0.37–0.44)	0.42 (0.38–0.46)	0.40 (0.37–0.45)	n.s.
	**BAIB**	0.98 (0.79–1.22)	1.51 (1.22–1.95)	2.41 (1.80–2.72)	<0.001	0.94 (0.74–1.21)	1.12 (0.88–1.38)	1.38 (0.96–1.89)	<0.001
	**ADMA**	0.37 (0.34–0.42)	0.38 (0.34–0.42)	0.36 (0.34–0.43)	n.s.	0.37 (0.34–0.41)	0.38 (0.34–0.42)	0.37 (0.34–0.42)	n.s.
	**L-arginine**	72.7 (57.4–96.4)	80.1 (61.2–116.3)	94.0 (70.0–146.8)	n.s.	74.3 (56.5–98.5)	71.5 (60.2–95.4)	82.8 (66.7–107.5)	n.s
**Urine (µmol/g creatinine)**		n = 347	n = 49	n = 4		n = 239	n = 143	n = 18	
	**SDMA**	35.9 (31.6–42.0)	37.3 (32.7–43.4)	33.4 (26.8–37.4)	n.s.	35.7 (32.1–41.1)	37.3 (31.0–42.7)	33.4 (30.9–44.0)	n.s.
	**BAIB**	49.4 (31.6–75.3)	165 (83.5–280)	863 (404–1246)	<0.001	47.0 (28.3–75.3)	65.5 (42.4–105)	125 (57.8–263)	<0.001
	**ADMA**	32.1 (27.1–38.1)	30.4 (27.2–37.1)	25.5 (20.9–27.8)	n.s.	31.6 (27.2–37.2)	32.2 (26.4–38.6)	29.9 (26.6–39.7)	n.s.
	**L-arginine**	14.0 (10.8–18.7)	13.6 (10.7–19.0)	12.4 (10.2–13.0)	n.s.	14.0 (10.7–19.0)	13.8 (11.0–17.4)	14.3 (10.7–30.3)	n.s.
**Creatinine**									
	**Serum (mg/dL)** †	0.8 (0.7–0.9)	0.8 (0.7–0.9)	1.0 (0.9–1.0)	<0.05	0.8 (0.7–0.9)	0.8 (0.7–0.9)	0.8 (0.7–0.9)	n.s.
	**Urine (mg/dL)**	86 (37–146)	71 (37–134)	144 (99–169)	n.s.	80 (34–147)	89 (38–139)	117 (38–185)	n.s.
	**Clearance (ml/min)** † ‡	123 (109–142)	125 (102–144)	120 (109–127)	n.s.	123 (109–141)	123 (109–144)	112 (102–140)	n.s.

Median values (25–75% percentile) are shown. The Kruskal-Wallis test was used for statistical analysis.

* Plasma concentrations of methylarginines, BAIB and L-arginine were determined for 395 volunteers.

† Serum concentrations of creatinine were determined for 390 volunteers.

‡ Cockgroft-Gault formula was used for calculation of creatinine clearance.

ADMA, asymmetric dimethylarginine; *AGXT2*, *alanine-glyoxylate aminotransferase 2*; BAIB, β-aminoisobutyrate; Hetero, heterozygous for minor allele; Homo, homozygous for minor allele; SDMA, symmetric dimethylarginine; WT (wild-type), homozygous for major allele.

Genotyping of the *AGXT2* SNP rs16899974 (c.1492G>T; p.Val498Leu) revealed a MAF of 0.22 (table 3 and table S1). In subjects heterozygous or homozygous for the minor allele BAIB concentrations were significantly higher by 19% and 47%, respectively, in plasma and by 40% and 166%, respectively, in urine (Kruskal-Wallis test: both p<0.001; table 3 and figure 1). However, the amino acid exchange p.Val498Leu was not associated with differences in plasma or urinary SDMA (table 3 and figure 1), ADMA or L-arginine concentrations (table 3).

### 4.3 *AGXT2* Haplotype Analysis and Additive Effects of the SNPs

For both *AGXT2* SNPs, allele frequencies did not significantly deviate from the Hardy-Weinberg equilibrium (rs37369 χ^2^ = 2.21 [p<0.05]; rs16899974 χ^2^ = 0.34 [p<0.05]). Haplotype analysis revealed also that both *AGXT2* SNPs, which have a distance of approximately 38 kbp on chromosome 5, are not significantly linked to each other (D’: 0.311; LOD: 2.20; r^2^∶0.026). This result was similar to the data of the International HapMap Project (D’: 0.452; LOD: 3.02; r^2^∶0.072) based on the analysis of the CEU panel (Utah residents with Northern and Western European ancestry from the CEPH [Centre d’Etude du Polymorphisme Humain] collection).

Our data indicate an independent and additive effect of both *AGXT2* SNPs with respect of accumulation of BAIB in plasma and urine. In two-way ANOVA models of log-transformed data both SNPs were independently associated with BAIB concentrations while the rs37369*rs16899974 interaction term was not significant (p<0.001, p = 0.0198 and p = 0.665, respectively, for rs37369, rs16899974 and the rs37369*rs16899974 interaction for BAIB in plasma, and p<0.001, p<0.001 and p = 0.376, respectively, for rs37369, rs16899974 and the rs37369*rs16899974 interaction for creatinine-indexed BAIB in urine). The increase of the number of minor alleles of either *AGXT2* SNP resulted in additional and significant accumulation of BAIB in plasma and urine (table 4). For SDMA only rs37369 was retained as significant in the two-way analyses. The two-way models for ADMA and L-arginine were not significant for either SNP.

**Table 4:** *A*nalysis of both determined *AGXT2* SNPs with regard to plasma (in µmol/L) and urinary (in µmol/g creatinine) BAIB concentrations.

Plasma BAIB	*AGXT2* rs16899974	*AGXT2* rs37369			
		WT (c.418 GG)	Hetero (c.418 GA)	Homo (c.418 AA)	
	**WT** (c.1492 GG)	0.92 (0.74–1.13); n = 218	1.43 (1.12–1.83); n = 20	2.51; n = 1	p<0.001
	**Hetero** (c.1492 GT)	1.05 (0.86–1.29); n = 114	1.55 (1.16–1.83); n = 22	2.31 (1.63–2.78); n = 3	p<0.001
	**Homo** (c.1492 TT)	1.08 (0.90–1.60); n = 13	2.03 (1.67–2.39); n = 4	/	p = 0.011
		p<0.001	p = 0.172	n.d.	
**Urinary BAIB**	***AGXT2*** ** rs16899974**	***AGXT2*** ** rs37369**			
		**WT (c.418 GG)**	**Hetero (c.418 GA)**	**Homo (c.418 AA)**	
	**WT** (c.1492 GG)	44.1 (27.9–67.9); n = 218	151 (83.1–230); n = 20	1036; n = 1	p<0.001
	**Hetero** (c.1492 GT)	58.5 (38.6–89.1); n = 116	167 (76.9–251); n = 24	689 (309–1316); n = 3	p<0.001
	**Homo** (c.1492 TT)	111 (51.1–140); n = 13	374 (292–587); n = 5	/	p<0.001
		p<0.001	p = 0.042	n.d.	

Median values (25–75% percentile) are shown. Both SNPs were significant for plasma and urinary BAIB in two-way ANOVA models using log-transformed data. The p values in the last rows and lower columns are based on nested ANOVA tests of log-transformed data (significant if p<0.025).

*AGXT2*, *alanine-glyoxylate aminotransferase 2*; BAIB, β-aminoisobutyrate; Hetero, heterozygous for minor allele; Homo, homozygous for minor allele; n.d., not determined; WT (wild-type), homozygous for major allele.

### 4.4 Correlation of ADMA, SDMA, BAIB and Creatinine Concentrations

In blood as well as in urine, SDMA concentrations were strongly and positively correlated to BAIB and creatinine concentrations (table 5). In case of plasma and urinary ADMA concentrations, the correlation with BAIB concentrations in plasma and urine was weaker but still significant, while no significant correlation was observed for plasma ADMA and creatinine concentrations in serum or urine.

**Table 5:** Correlation of ADMA, SDMA, BAIB and creatinine concentrations measured in blood and urine.

		Plasma			Serum	Urinary			
		ADMA	SDMA	BAIB	Crea	ADMA	SDMA	BAIB	Crea
**Plasma ADMA**	rho value		0.310	0.145	-0.012	0.182	0.091	0.145	0.076
	p value		<0.001	<0.01	n.s.	<0.001	n.s.	<0.01	n.s.
**Plasma SDMA**	rho value	0.310		0.466	0.506	0.154	0.233	0.265	0.247
	p value	<0.001		<0.001	<0.001	<0.01	<0.001	<0.001	<0.001
**Plasma BAIB**	rho value	0.145	0.466		0.246	0.139	0.188	0.617	0.173
	p value	<0.01	<0.001		<0.001	<0.01	<0.001	<0.001	<0.01
**Serum Crea**	rho value	-0.012	0.506	0.246		0.150	0.277	0.209	0.325
	p value	n.s.	<0.001	<0.001		<0.01	<0.001	<0.001	<0.001
**Urinary ADMA**	rho value	0.182	0.154	0.139	0.150		0.981	0.698	0.951
	p value	<0.001	<0.01	<0.01	<0.01		<0.001	<0.001	<0.001
**Urinary SDMA**	rho value	0.091	0.233	0.188	0.277	0.981		0.720	0.971
	p value	n.s.	<0.001	<0.001	<0.001	<0.001		<0.001	<0.001
**Urinary BAIB**	rho value	0.145	0.265	0.617	0.209	0.698	0.720		0.688
	p value	<0.01	<0.001	<0.001	<0.001	<0.001	<0.001		<0.001
**Urinary Crea**	rho value	0.076	0.247	0.173	0.325	0.951	0.971	0.688	
	p value	n.s.	<0.001	<0.01	<0.001	<0.001	<0.001	<0.001	

For calculations the Spearman’s test was used.

ADMA, asymmetric dimethylarginine; BAIB, β-aminoisobutyrate; Crea, creatinine; n.s., not significant; SDMA, symmetric dimethylarginine.

### 4.5 *In vitro* Evidence that *AGXT2* rs37369 Results in Reduced Enzyme Activity

**Figure 2:**
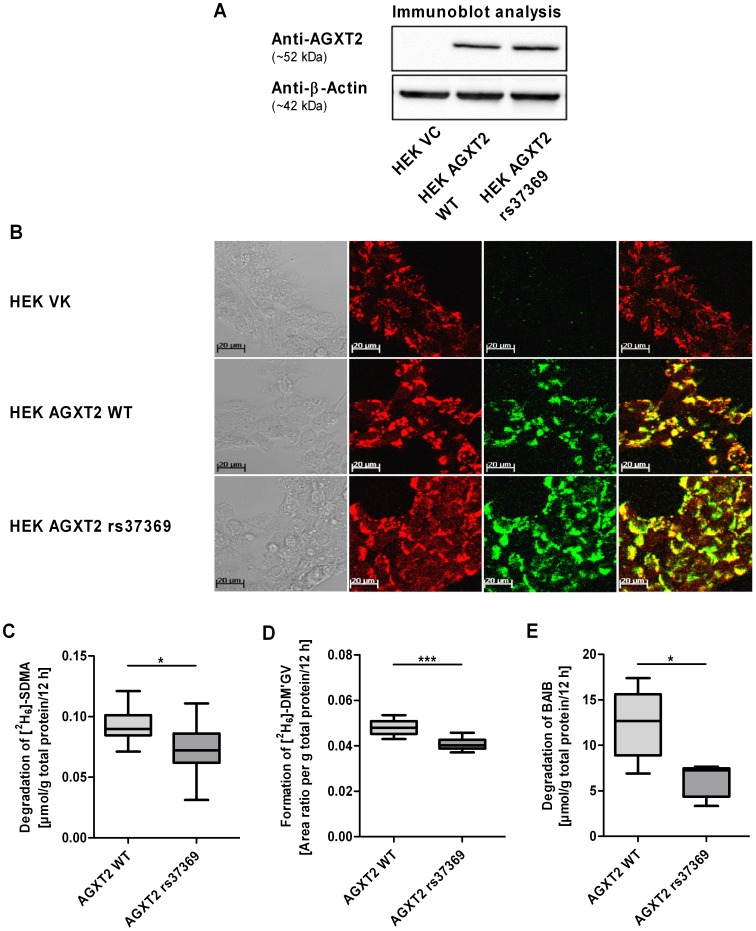
Characterization of HEK cell lines overexpressing human wild-type and mutant (rs37369, p.Val140Ile) AGXT2 protein. HEK cells overexpressing human wild-type (AGXT2 WT) and mutant (AGXT2 rs37369) protein showed similar AGXT2 protein expression levels determined by immunoblot (A, representative immunoblot; 20 µg total protein per line) and immunofluorescence (B) analysis. AGXT2 was localized to mitochondria in both cell lines. Cells transfected with the empty vector (VC) were used as negative control. Enzyme activity of mutant AGXT2 indicated significant reduction of [^2^H_6_]-SDMA degradation (C; each n = 10), [^2^H_6_]-DM’GV formation (D; each n = 10) and BAIB degradation (E; each n = 5) compared to wild-type protein normalized to total protein. Area ratio is defined as [^2^H_6_]-DM’GV/DM’GV. Values are median ±1.5 IQR. Unpaired 2-tailed Student’s *t* test (* p<0.05, *** p<0.001) was used for statistical analysis. AGXT2, alanine-glyoxylate aminotransferase 2; DM’GV, (N,N’-dimethyl-guanidino)valeric acid (AGXT2-dependent metabolite of SDMA); HEK cells, human embryonic kidney 293 cells; VC, vector control; WT, wild-type.

Cell lines stably overexpressing wild-type and mutant AGXT2 protein were established to determine functional consequences of the amino acid exchange p.Val140Ile resulting from *AGXT2* SNP rs37369, which was associated with the largest differences in BAIB and SDMA plasma concentrations *in vivo*. Immunoblot analysis confirmed that protein expression of cell lines overexpressing wild-type or mutant protein (p.Val140Ile) was similar to each other (arbitrary units: 9.7±0.2 versus 10.5±1.2, respectively, normalized to β-actin expression; p>0.05) as shown in figure 2A. Immunofluorescence analysis indicated intracellular localization of AGXT2 to mitochondria [26] in both cell lines (figure 2B). Cells transfected with the empty vector were used as negative control.

In order to assess the enzyme activity, samples of cell lysates from HEK cells overexpressing wild-type AGXT2, mutant AGXT2 or the empty vector were incubated with BAIB or [^2^H_6_]-SDMA. In preliminary experiments lysates of cells transfected with the empty vector indicated almost no enzyme activity with regard to the degradation of BAIB or [^2^H_6_]-SDMA as well as the formation of the AGXT2-dependent metabolite [^2^H_6_]-DM’GV compared to lysates of cells overexpressing wild-type AGXT2.

When comparing activity of wild-type and mutant AGXT2 a significant reduction of enzyme activity was observed (figure 2C–E). Degradation of the AGXT2 substrate [^2^H_6_]-SDMA (figure 2C) and formation of the metabolite [^2^H_6_]-DM’GV (figure 2D) were significantly reduced by 20% (*t* test: p<0.05) and 16% (*t* test: p<0.001), respectively, in lysates of cells overexpressing mutant protein compared to wild-type. Moreover, degradation of BAIB was decreased by 43% as compared to wild type enzyme (figure 2E, *t* test: p<0.05).

### 4.6 *In silico* Modeling of *AGXT2* SNPs Revealed Reduced AGXT2 Stability and Substrate Binding

**Figure 3:**
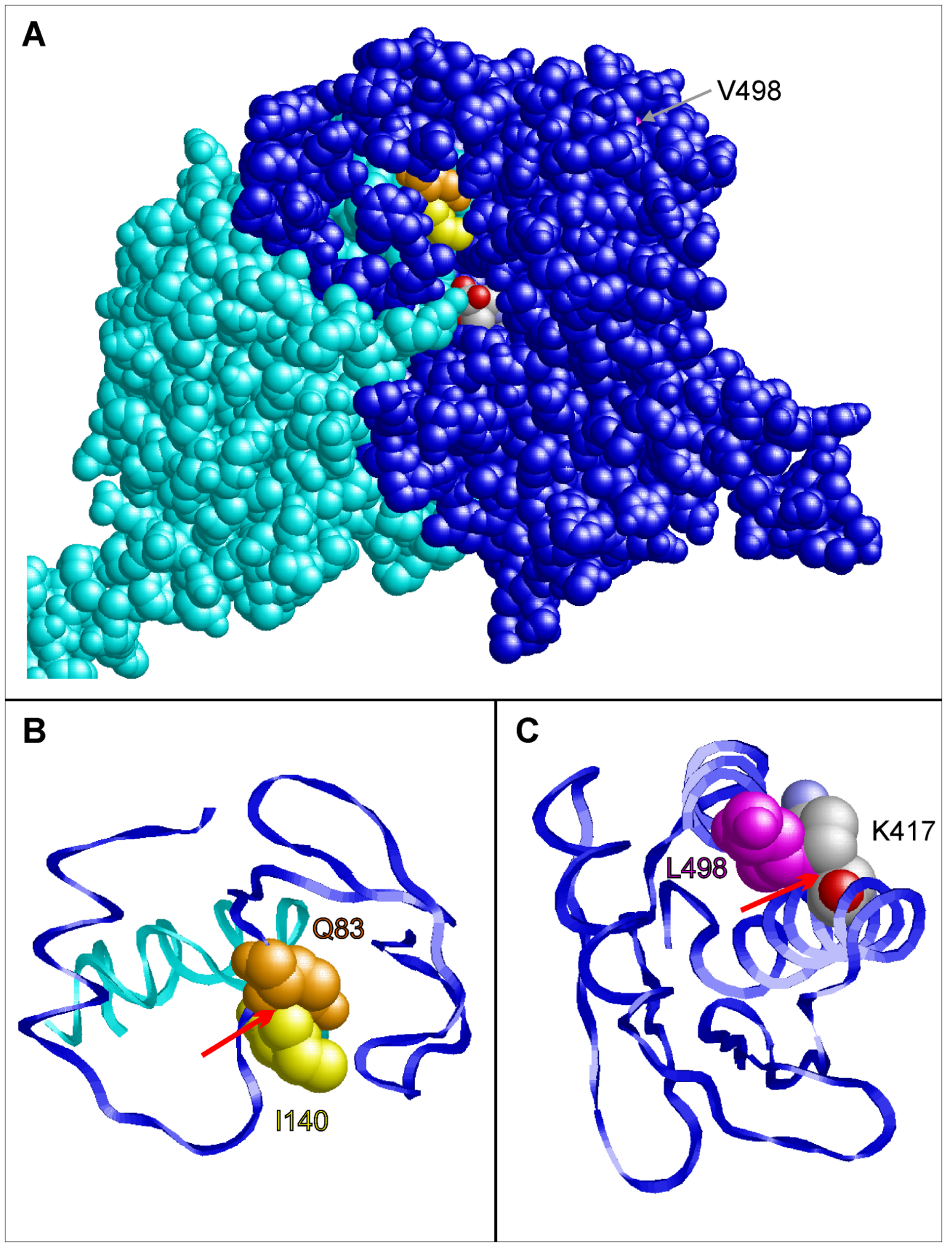
Prediction of the phenotypic effects of the coding SNPs rs37369 (p.Val140Ile) and rs16899974 (p.Val498Leu) in AGXT2 using structural information from *in silico* homology modeling. (A) Three-dimensional model of the AGXT2 (V140I) dimer showing the subunits A (blue) and B (cyan) in space-filled presentation. Residues Q83 (chain A), I140 (chain B), and V498 (chain A) are colored in orange, yellow and magenta, respectively. The β-aminoisobutyrate substrate of subunit A is colored according to the atom types. This view shows that V498 is buried in the interior of the molecule, whereas I140 of chain B is located close to the substrate binding site of subunit A. (B) Enlargement of the site of the V140I mutation. Residues 60–120 of chain A (blue) and residues 120–150 of chain B (cyan) are shown in ribbon presentation. A red arrow denotes a clash between the side chains of I140 (chain B; yellow) and Q83 (chain A; orange) that is not observed for V140 in the wild-type. (C) Enlargement of the C-terminal residues 400–500 showing the effect of a V498L mutation. The larger side chain of L498 forms steric clashes with the methylene groups of the K417 side chain (denoted by a red arrow).

To find a structural explanation of the experimentally observed effects of the V140I (*AGXT2* rs37369) and V498L (*AGXT2* rs16899974) mutations, 3D molecular models were constructed for wild-type and mutant AGTX2. Residue 140 is located in a loop of the protein that is close to the substrate binding site (figure 3A). In the wild-type protein, this residue forms tight interactions with an adjacent glutamine (Q83) of the second subunit. Due to the larger side chain in the V140I mutant, clashes are observed between I140 and Q83 (figure 3B). This I140-Q83 clash is predicted to have an effect on dimer stability and on substrate access to the active site and thereby offers an explanation for the experimentally observed reduced enzymatic activity.

V498 is located in a helix in the C-terminal part of AGXT2 (figure 3C). As a consequence of the V498L mutation, the larger L498 side chain forms steric clashes with the methylene groups of the K417 side chain. This is expected to result in local structural rearrangement and might also cause a decreased stability of the enzyme. In line with its location far apart from the active site, the experimentally observed changes in enzymatic activity are less pronounced than those detected for the V140I mutation.

## Discussion

Based on data from 400 healthy volunteers as well as *in vitro* and *in silico* experiments the present study provides strong evidence that metabolism of SDMA and BAIB is intra-individually affected by the same coding SNPs of *AGXT2* which underlie the common metabolic trait of hyper-β-aminoisobutyric aciduria.

In 2011 an association of the *AGXT2* SNP rs37369 with plasma SDMA concentrations [27] and urinary BAIB excretion [15], [28] was noted in three independent genome-wide association studies (GWAS)-based investigations. Furthermore, during finalization of this manuscript another coding *AGXT2* SNP (rs37370) was linked to elevated plasma BAIB concentrations in the Framingham Heart Study (FHS) [29] and some years ago to elevated urinary BAIB concentrations in a human metabolome study [28] indicating that *AGXT2* SNPs can result in BAIB accumulation. The present study now connects and extends these different lines of evidence by showing that SDMA and BAIB metabolism are intra-individually linked to the same amino acid exchange (p.Val140Ile) in the AGXT2 protein which results in decreased enzyme activity. In several cohorts it could be demonstrated that 40% of the Asian population are high BAIB excretors with an underlying genetic background [30], [31]. Given the similar prevalence of *AGXT2* SNP rs37369 in several Asian populations (MAF: 0.57) we propose that this SNP is responsible for the BAIB accumulation [10]. In addition we show that a second *AGXT2* SNP (rs16899974) may aggravate the effect of rs37369. The additive effect on BAIB concentrations, the different MAF as well as haplotype analysis indicate that the two polymorphisms seem not to be linked to each other as haplotype. In a previous study Kontani and colleagues [10] determined a 50-fold lower K_m_ value of rat Agxt2 using BAIB as substrate compared to SDMA. The clear accumulation of BAIB in plasma (146%) and urine (1661%) associated with the *AGXT2* SNP rs37369 suggests that in humans BAIB is mainly metabolized by AGXT2. If AGXT2 is indeed the main enzyme in degrading BAIB and possesses high affinity to BAIB as substrate, elevated BAIB concentrations could function as a good indicator for AGXT2 enzyme activity.

It has been speculated that SDMA could serve as a sensitive marker of renal function as its plasma concentrations increases in a similar fashion as serum creatinine when renal function is impaired [7], [32]. Our data indeed show a strong correlation of plasma SDMA and creatinine in relatively young healthy volunteers which is comparable to that previously observed in older adults [33]. Impaired renal function is a well established risk factor for cardiovascular events and total mortality. Therefore, it was and is tempting to attribute the association of SDMA and mortality to the fact that SDMA simply serves as an indicator of impaired renal function. This cannot explain, however, why SDMA remains a predictor of mortality when adjusting for renal function [34], [35]. It is possible that SDMA itself can act as an uremic toxin via inhibition of L-arginine uptake [6] or activation of store-operated Ca^2+^-channels [36]. Here, the present data indicate that metabolism of SDMA may not be as negligible as frequently assumed. Especially, when renal function is within the healthy range common *AGXT2* SNPs should be taken into account, too. This brings the attention to the fact that AGXT2 has several other substrates besides SDMA including BAIB. Particularly the link to hyper-BAIB aciduria may offer alternative explanations for the association of SDMA and mortality. The long term prognostic significance of hyper-BAIB aciduria and the corresponding *AGXT2* SNPs is currently under investigation.

Caplin et al. [14] observed elevated ADMA and SDMA plasma concentrations in *Agxt2* deficient mice. We could show that BAIB infusion inhibits AGXT2-mediated degradation of SDMA and ADMA in mice [20]. However, in contrast to the studies in rodents [12], [14], [20] and patients with kidney transplants [14] our data suggest that in healthy humans the impact of AGXT2 activity on plasma concentrations may be more limited for ADMA than for SDMA. This may be explained by the fact that ADMA (in contrast to SDMA) can also be metabolized by DDAH1 which may be able to compensate deficient metabolism by AGXT2. However, the significant correlation of ADMA and BAIB we observed in plasma and urine still suggests shared routes of elimination (i.e. metabolism and transport). This finding may be of relevance for the interpretation of recent GWAS data showing an association of AGXT2 and hypertension [14] as well as heart rate variability [37]. Caplin et al. [14] also reported that *Agxt2* KO mice are associated with a significant increase in blood pressure (∼50%) suggesting a possible role for AGXT2 in cardiovascular diseases. Relying on the observations in mice the association was largely attributed to a possible impact of the *AGXT2* SNP as data on plasma concentrations of ADMA and SDMA were not available for that study. Based on the data of the current study ADMA-related impairment of nitric oxide synthases has become an unlikely (but still not impossible) explanation and alternative AGXT2 substrates should be explored as possible mediators of hypertension associated with *AGXT2* SNPs.

Very recent GWAS data connecting *AGXT2* SNPs to homoarginine serum concentrations [38] may offer yet another explanation as homoarginine has been identified as an independent risk marker for mortality, which may also be related to NO generation. However, in our cohort we did not observe any significant association of *AGXT2* SNPs with homoarginine plasma concentrations (data not shown).

In conclusion, this study shows for the first time that the *AGXT2* SNP rs37369 (p.Val140Ile), which results in hyper-BAIB aciduria, is associated with elevated plasma concentrations of BAIB and SDMA in healthy humans. Considering that plasma SDMA concentrations are linked to adverse events in cardiovascular diseases and renal failure polymorphisms within the *AGXT2* gene could have further implications in disease progression and require further investigations.

## Supporting Information

Figure S1 **Synthesis of DM’GV used as reference in HPLC-MS/MS.** α-Keto-δ-(N,N’-dimethylguanidino)valeric acid (DM’GV) was prepared from symmetric dimethylarginine (SDMA) according to a procedure reported by Klein et al. [1]. Treatment of SDMA with trifluoroacetic acid anhydride hereby leads to the formation of a 2-trifluoromethyl-3-oxazolin-5-one intermediate, which undergoes cleavage to trifluoroacetaldehyde and keto acid under strongly basic aqueous conditions. 1.Klein C, Schulz G, Steglich W (1983) Conversion of ω-guanidino- and ω-ureido-α-amino acids into α-keto acids and heterocycles derived therefrom. Liebigs Ann. Chem.: 1623–1637.
(TIF)

Table S1 Genotype distribution of *AGXT2* rs37369 and rs16899974 in the cohort of 400 healthy volunteers.
(DOCX)
