# Supplementary material for: Alanine-glyoxylate aminotransferase 2 (AGXT2) Polymorphisms Have Considerable Impact on Methylarginine and β-aminoisobutyrate Metabolism in Healthy Volunteers
Source: PLoS One. 2014 Feb 24;9(2):e88544. doi: 10.1371/journal.pone.0088544 (PMC3933329; doi:10.1371/journal.pone.0088544)
Supplement: Table S1 — Genotype distribution of AGXT2 rs37369 and rs16899974 in the cohort of 400 healthy volunteers. (DOCX) [file pone.0088544.s002.docx]

**Table S1. Genotype distribution of *AGXT2* rs37369 and rs16899974 in the cohort of 400 healthy volunteers.**

| ***AGXT2* rs16899974** | | ***AGXT2* rs37369** | | |
| --- | --- | --- | --- | --- |
|  |  | **WT** (c.418 GG) | **Hetero** (c.418 GA) | **Homo** (c.418 AA) |
|  |  | 347 (86.75 %) | 49 (12.25 %) | 4 (1 %) |
| **WT** (c.1492 GG) | 239 (59.75 %) | 218 (54.5 %) | 20 (5.0 %) | 1 (0.25 %) |
| **Hetero** (c.1492 GT) | 143 (35.75 %) | 116 (29.0 %) | 24 (6.0 %) | 3 (0.75 %) |
| **Homo** (c.1492 TT) | 18 (4.5 %) | 13 (3.25 %) | 5 (1.25 %) | 0 (0 %) |

Values are shown as number of volunteers (genotype frequency in %).

*AGXT2* rs37369: c.418G>A; p.Val140Ile; Hardy-Weinberg equilibrium χ^2^=2.21 (p>0.05)

*AGXT2* rs16899974: c.1492G>T; p.Val498Leu; Hardy-Weinberg equilibrium χ^2^=0.34 (p>0.05)

*AGXT2*, *alanine-glyoxylate aminotransferase 2*; Hetero, heterozygous for minor allele; Homo, homozygous for minor allele; WT (wild-type), homozygous for major allele.
